# 110 Implementation of 3D Technology for Management of Facial Scars Across the Spectrum of Care

**DOI:** 10.1093/jbcr/iraf019.110

**Published:** 2025-04-01

**Authors:** Andrea Mc Kittrick, Jason Brown

**Affiliations:** Royal Brisbane and Women’s Hospital; Royal Brisbane and Women’s Hospital

## Abstract

**Introduction:**

Burn injuries to the face have long been described to have significant physical and psychological impacts. The management of facial burns across the spectrum of care is complex due to the anatomical structures and shape. No single surface is linear, and each surface is dynamic allowing for function, expression and animation. The use of 3D scanning to fabricate moulds for transparent facial orthosis (TFO) in not new however to date no study has explored how early this technology can be used in the management of facial burns, and whether a point-of-care manufacturing workflow can be effective.

**Methods:**

This was a prospective cohort study. Ethical approval was granted by the local ethics and governance committee. All patients (> 18 years or >14 years with parental consent) with facial burns requiring wound management with dressings were considered. Screening for eligibility occurred upon admission to the ICU or the burn ward. Enrolled participants were scanned using a wireless, handheld, portable 3D scanner (Artec Leo) once the wound bed had been cleaned. The occupational therapist reviewed each 3D scan to determine which scan could be used to create the TFO. The best 3D scan was converted to a 3D model by the research engineer using the Artec Studio software. The 3D model was used as a template to create the TFO by research engineer and the occupational therapist.

**Results:**

n= 15 were enrolled in the study. 12=male 3= female. Mechanism of injury flame n= 15. Mean TBSA = 45.2%. Burn Depth on the face ranged from partial thickness to deep dermal. n= 15 underwent surgical debridement of their facial burns. Allograft was applied in 80% of cases. n=1 passed away. 30% had their 3D scan completed in ICU. Progression of oedema in the first 24-48 hours post burn was the primary barrier to capturing the 3D scan. All other 3D scans were completed post ICU step down to the burns ward. n=1 adverse event was recorded, a pressure injury on the bridge of a nose. No other adverse effects were recorded. 33% of participants required fabric compression masks in addition to their TFO. All participants received standard care of scar massage, range of motion, splinting and silicone therapies.

**Conclusions:**

Early 3D scanning is rapid, non-invasive and produces a high-quality 3D model suitable for producing an accurate TFO. This 3D scanner is suitable to use on wards and in the ICU as infection control is achieved using sanitising wipes. Furthermore, having a digital model of the individuals face is highly useful for inspection of the final mask design and can be used to quantitatively track changes in scarring over time.

**Applicability of Research to Practice:**

The workflow identified this study is a practical approach that can provide benefits to patient care; however, it is critically important that the scanning device, software, quality assurance methods and technical experience of the engineering staff are appropriate to ensure a safe and effective TFO device.

**Funding for the Study:**

N/A

